# Lack of Host Specialization on Winter Annual Grasses in the Fungal Seed Bank Pathogen *Pyrenophora semeniperda*

**DOI:** 10.1371/journal.pone.0151058

**Published:** 2016-03-07

**Authors:** Julie Beckstead, Susan E. Meyer, Toby S. Ishizuka, Kelsey M. McEvoy, Craig E. Coleman

**Affiliations:** 1 Department of Biology, Gonzaga University, Spokane, Washington, 99258, United States of America; 2 USDA Forest Service, Rocky Mountain Research Station, Shrub Sciences Laboratory, Provo, UT 84606, United States of America; 3 Department of Plant and Wildlife Sciences, Brigham Young University, Provo, UT 84602, United States of America; Universita degli Studi di Pisa, ITALY

## Abstract

Generalist plant pathogens may have wide host ranges, but many exhibit varying degrees of host specialization, with multiple pathogen races that have narrower host ranges. These races are often genetically distinct, with each race causing highest disease incidence on its host of origin. We examined host specialization in the seed pathogen *Pyrenophora semeniperda* by reciprocally inoculating pathogen strains from *Bromus tectorum* and from four other winter annual grass weeds (*Bromus diandrus*, *Bromus rubens*, *Bromus arvensis* and *Taeniatherum caput-medusae*) onto dormant seeds of *B*. *tectorum* and each alternate host. We found that host species varied in resistance and pathogen strains varied in aggressiveness, but there was no evidence for host specialization. Most variation in aggressiveness was among strains within populations and was expressed similarly on both hosts, resulting in a positive correlation between strain-level disease incidence on *B*. *tectorum* and on the alternate host. In spite of this lack of host specialization, we detected weak but significant population genetic structure as a function of host species using two neutral marker systems that yielded similar results. This genetic structure is most likely due to founder effects, as the pathogen is known to be dispersed with host seeds. All host species were highly susceptible to their own pathogen races. Tolerance to infection (i.e., the ability to germinate even when infected and thereby avoid seed mortality) increased as a function of seed germination rate, which in turn increased as dormancy was lost. *Pyrenophora semeniperda* apparently does not require host specialization to fully exploit these winter annual grass species, which share many life history features that make them ideal hosts for this pathogen.

## Introduction

Generalist pathogens often play critical roles in natural plant communities. For example, the presence or absence of a generalist pathogen can determine the outcome of competition [1] or determine whether an invasive species displaces a native species [2]. Complex host-generalist pathogen interactions have frequently been demonstrated in tropical ecosystems [3–5], but are much less well-studied in temperate semi-deserts [6–7].

Pathogens that are generalists at the species level may be true generalists or they may be comprised of physiological races specific to a single host or subset of hosts. Most studies of host specialization have dealt with pathosystems in which host resistance is regulated through gene for gene interactions, and resistance and susceptibility are expressed qualitatively [8]. The role of host specialization in pathosystems that lack gene for gene recognition is much less well-studied [9]. It is more likely to be expressed as quantitative differences in aggressiveness on different hosts [10–11]. This type of specialization is more difficult to measure, as disease levels are more likely to vary as a function of inoculum load and environmental conditions as well as host and pathogen genotype. Host specialization is often accompanied by genetic differentiation that may be interpreted to be a consequence of diversifying selection, even if this is not directly demonstrable in associated pathogenicity tests [12]. Alternatively, pathogen population genetic structure among hosts could be due to other evolutionary processes, for example, founder effects or genetic drift.

Seed pathogens are important contributors to disease-related mortality in plant populations [13]. In this investigation, we focus on *Pyrenophora semeniperda*, a naturally-occurring ascomycete seed pathogen found primarily in the seed banks of the invasive annual grass *Bromus tectorum* (cheatgrass, downy brome) and other winter annual grasses. *Pyrenophora semeniperda* is a generalist that can infect seeds of multiple hosts [14–15]. Our primary question is whether strains of *P*. *semeniperda* from the seed banks of different weedy annual grasses exhibit host specialization. In a host range study including a wide range of grass species, Beckstead et al. [15] found that hosts more closely related to *B*. *tectorum* were more susceptible than distantly related hosts to *P*. *semeniperda* strains originating from *B*. *tectorum*, suggesting some level of specificity. In contrast, Beckstead et al. [6] demonstrated spillover of *P*. *semeniperda* from *B*. *tectorum* onto co-occurring native cool-season grasses, suggesting that host specialization was weak or absent.

**Fig 1:**
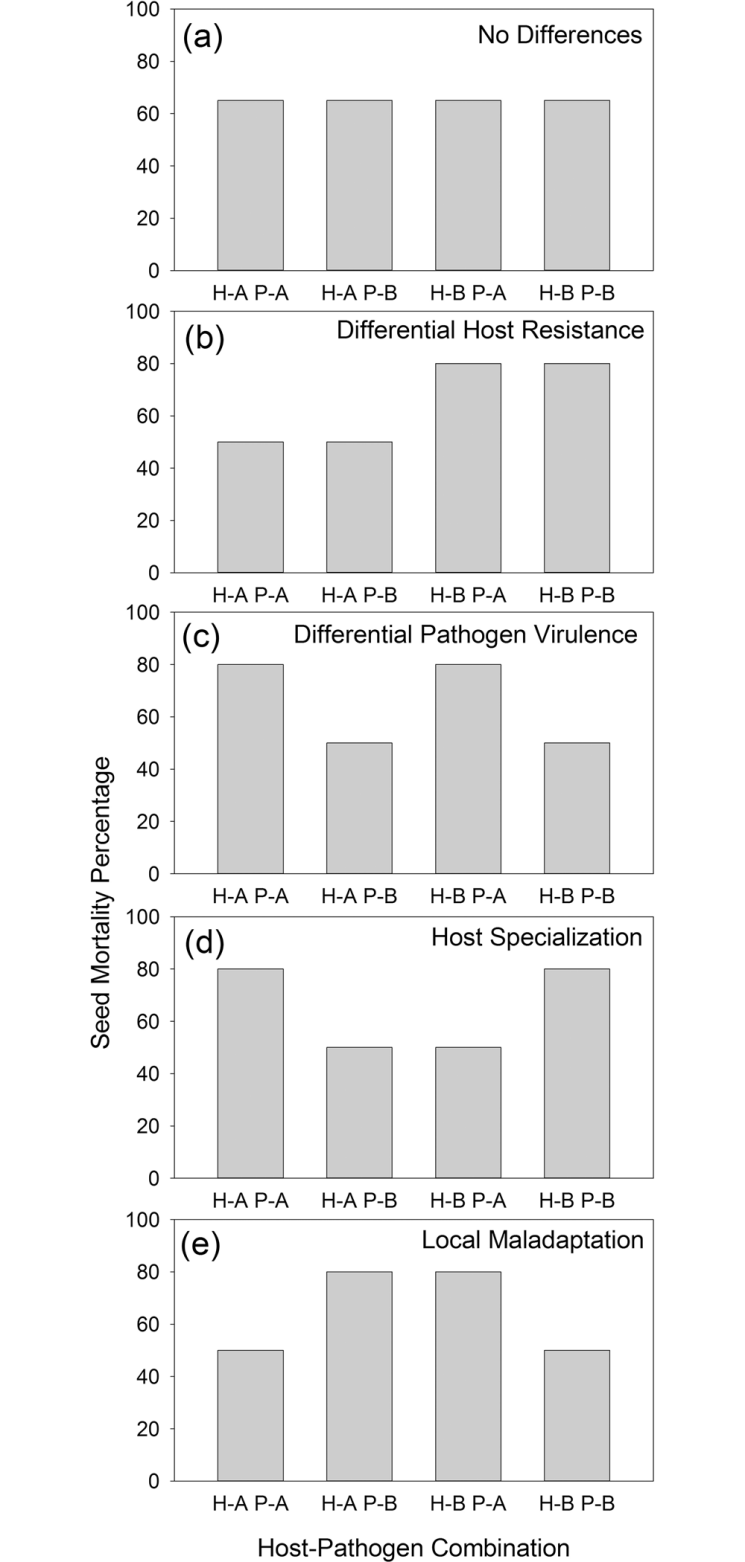
Hypothetical cross-inoculation predictions for hosts (Host-A species or Host-B species) and pathogens (Pathogen-A strain or Pathogen-B strain) demonstrating: (a) no difference, (b) difference in host resistance, (c) difference in pathogenicity, (d) pathogen host specialization, (e) pathogen local maladaptation.

Host-pathogen cross-inoculation experiments can result in at least five possible outcomes: no differential response, one host more resistant to both pathogen populations, one pathogen population more pathogenic on both hosts, host specialization (pathogen populations more pathogenic on hosts of origin), or local maladaptation (pathogen populations less pathogenic on hosts of origin; Fig 1a–1e). Although the majority of studies on multiple host-pathogen evolutionary outcomes have focused on parasite systems [16–17], there are many studies of generalist plant pathogens and their hosts. For example, Konno et al. [10] used cross-inoculation experiments to demonstrate that strains of *Colletotrichum anthrisci*, a generalist pathogen of woody plants, showed higher virulence when inoculated onto the host of origin than when inoculated onto a novel host, demonstrating host specificity. Kniskern et al. [9] found evidence for local maladaptation in the generalist bacterial plant pathogen *Pseudomonas syringae* in an experiment with strains from different hosts on inbred lines of *Arabidopsis thaliana*. Carlsson-Graner [18] found that infection levels did not differ in cross-inoculation experiments with *Microbotryum violaceum* on two sympatric hosts, indicating neither local adaptation nor maladaptation.

A second question addressed by this study is whether the pattern of resistance and tolerance to *P*. *semeniperda* observed for *B*. *tectorum* [15, 19] is evident in other weedy winter annual grasses. Roy and Kirchner [20] described resistance as the ability of the host to avoid infection, as opposed to tolerance, which is the ability of the host to tolerate the consequences of infection. *Bromus tectorum* seeds are highly susceptible to infection by *P*. *semeniperda* whether dormant or nondormant and therefore have low resistance to infection, but their tolerance to infection varies as a function of dormancy status, with nondormant seeds suffering much lower mortality than dormant seeds because of their ability to germinate rapidly and thereby escape death. Beckstead et al. [15] found a similar pattern across species, i.e., among equally susceptible species, those with slower germination rates were more likely to suffer mortality than species that germinated more quickly. The within-species change in tolerance as a function of dormancy status was quantified only for *B*. *tectorum*, but was postulated to occur in other weedy annual grasses that lose dormancy through after-ripening.

Our experimental approach was designed to test the following hypotheses: (1) *P*. *semeniperda* populations from different winter annual grass species exhibit population genetic structure that includes a significant among-population component, i.e., pathogen populations show genetic differentiation on different host species, (2) Pathogen populations from different winter annual grass species exhibit host specialization, i.e., they cause higher levels of disease on their host of origin than on a novel host, (3) Seeds of winter annual grass species exhibit high susceptibility to infection by co-occurring *Pyrenophora semeniperda* strains whether dormant or nondormant, but are intolerant of infection (i.e., killed) only when dormant.

## Materials and Methods

### Field sample collections

The five host species included were: *B*. *tectorum*, *Bromus diandrus*, *Bromus rubens*, *Bromus arvensis* (formerly *Bromus japonicus*), and *Taeniatherum caput-medusae*. Seed and soil seed bank collections were made from May-July 2012. All study sites contained near-monocultures of the annual grass collected. Seeds were collected by hand-stripping and combined into a bulk collection for each species. Soil seed bank samples were made with steel cans 6–10 cm diameter and 2–4 cm deep. For two of five host species, seed and soil seed bank collections were from the same site; for the remaining species, collections were made at two nearby sites (Table 1). Lower Zion Canyon and Spanish Fork collections were made on private land with landowner permission. All remaining collections were made on open access public land with no permit required for collection. No protected or endangered species were collected. In cases where host and pathogen populations were collected from different locations, the distance between the two locations was generally <100 km.

**Table 1:** Location information for host seed and *P*. *semeniperda* collection sites.

Host Species^1^	Code	Location	State	Latitude/Longitude	Elev. (m)
**Pathogen Populations**					
*Bromus tectorum* (Cheatgrass)	BRTE	Ten Mile Creek	UT	41.86488–113.13594	1540
		Whiterocks	UT	40.32818–112.77816	1440
*Bromus diandrus* (Ripgut brome)	BRDI	Lower Zion	UT	37.19728–112.99006	1215
		Canyon			
*Bromus rubens* (Red brome)	BRRU	West of Hurricane	UT	37.17341–113.34776	955
*Bromus arvensis* (Japanese brome)	BRAR	Fishtrap	WA	47.39115–117.83991	1690
*Taeniatherum caput-medusae* (Medusahead)	TACA	North of Bliss	ID	42.99860–114.96619	1090
**Host Populations**					
*Bromus tectorum* (Cheatgrass)	BRTE	Spanish Fork	UT	40.06746–111.63215	1430
*Bromus diandrus* (Ripgut brome)	BRDI	Castle Cliff	UT	37.07090–113.89095	1190
*Bromus rubens* (Red brome)	BRRU	North of Littlefield	AZ	36.99761–113.92078	780
*Bromus arvensis* (Japanese brome)	BRAR	Fishtrap	WA	47.39115–117.83991	1690
*Taeniatherum caput-medusae* (Medusahead)	TACA	North of Bliss	ID	42.99860–114.96619	1090

^1^ Nomenclature based on USDA, NRCS. 2014. The PLANTS Database (http://plants.usda.gov, 21 January 2014). National Plant Data Center, Baton Rouge, LA USA.

All seed lots showed high viability (> 95%). Seeds were stored at room temperature for less than two weeks prior to placement into temperature treatments to retain primary dormancy (-4°C) or to break dormancy through after-ripening (35°C; [21]). Dormancy status was evaluated through periodic monitoring of germination rate for seeds of each species. When germination was initiated by day 2–4 at 25°C, seeds were considered to have lost primary dormancy.

### Pathogen strain isolations

Soil seed bank samples were screened on a soil sieve, and field-killed seeds with stromata were extracted by hand. Thirty *P*. *semeniperda*-killed seeds with distinctive stromata (length 2–5 mm) were selected from each grass species. Fungal cultures were prepared for genetic characterization by plating surface-sterilized field-collected stromata onto PDA (potato dextrose agar), single-sporing onto V-8 agar, subculturing the resulting monoconidial cultures in PDB (potato dextrose broth), and drying the resulting mycelial biomass for DNA extraction (see [22] for methods).

For conidial production, pure monoconidial cultures on V8 agar of a subset of six genetically distinct strains (based on molecular marker fingerprints; see next section) selected from the original isolate collection for each host were transferred to MAM (modified alphacel medium; [23]) and placed under fluorescent and UV light at room temperature to induce sporulation. Conidia were produced, harvested, and tested for germination as described in [24]. They were air-dried and stored in glassine envelopes at room temperature until experiment initiation a few weeks later.

### Pathogen population genetic characterization

DNA was extracted from the dried mycelial cultures of each isolate as described in [22]. Sample numbers were: *B*. *tectorum* n = 21, *B*. *diandrus* n = 21, *B*. *arvensis* n = 19, *B*. *rubens* n = 23, *T*. *caput-medusae* n = 24. ITS (internal transcribed spacer sequences from ribosomal DNA) genotyping was carried out as described in [22]. Microsatellite (SSR or single sequence repeat) genotyping was carried out using seven polymorphic microsatellite loci developed from 454 pyrosequencing data [25]. Microsatellite marker primers and methods are described in Meyer et al. [26]. Fragment analysis was carried out on an ABI 3100 Genetic Analyzer (Applied Biosystems) and peak analysis was performed using ‘Peak Scanner Software v.1.0’ by Applied Biosystems.

Analysis of peak files from the microsatellite analysis revealed that many of the strains included in the study were heterokaryotic, i.e., they contained two genetically distinct types of nuclei in spite of their monoconidial origin. This is a common phenomenon in filamentous ascomycete fungi that results from hyphal fusion of vegetatively compatible but genetically distinct haploid mycelia [27]. For purposes of population genetic analyses based on microsatellite allele frequencies, each of these heterokaryotic strains was included as two haploid strains, effectively increasing sample size for the analysis. Heterokaryotic strains did not exhibit polymorphism at the ITS locus. These heterokaryotic strains were essentially functionally diploid individuals.

Allele frequency data from the two marker systems were analyzed separately. Arlequin 3.5 [28] was used to perform analysis of molecular variance for several alternative pathogen population structures, to calculate expected heterozygosity (gene diversity) for each pathogen population, and to generate a genetic distance matrix based on pairwise F_ST_ (the fixation index, a measure of population differentiation due to genetic structure [28]). The genetic distance matrix from each marker system was then used to generate a dendrogram of population relationships with the program Neighbor in the PHYLIP software package [29], using the UPGMA (Unweighted Pair Group Method with Arithmetic Mean) clustering protocol with other settings at default values. Because pathogen populations for this study were collected from a wide geographic area, a second analysis was performed using an ITS data set that included all pathogen populations in the present study along with three additional pathogen populations on *B*. *tectorum* from the earlier ITS study [22] that were in proximity to study populations from the other hosts (Cricket Mountain, Southern Region to compare with populations from *B*. *rubens* and *B*. *diandrus*; Cinder Cone Butte, Central Region to compare with the population from *T*. *caput-medusae*; and Fishtrap, Northern Region to compare with the population from *B*. *arvensis*). The objective was to determine whether the observed population differentiation was more likely to be explained by geographic distance or by host of origin.

### Cross-inoculation experiments

The experimental design for the cross-inoculation experiments to test for host specialization included four separate experiments with reciprocal inoculation of pathogen populations from *B*. *tectorum* and one alternate host species onto dormant seeds of these two species. The same treatment for *B*. *tectorum* pathogen strains inoculated onto the *B*. *tectorum* host was included as one of the four main treatments in all four experiments. Each pathogen population was represented by the six strains for which conidia were available (only four strains for *B*. *arvensis*). For each treatment combination (host species x strain), eight replicates of 25 inoculated seeds were included. The selected inoculum density (1:1000 dilution by weight) was obtained by diluting pure conidia with sterile reagent-grade talc (hydrated magnesium silicate). This inoculum load resulted in intermediate mortality of dormant *B*. *tectorum* seeds (40%) in an experiment with a gradient of inoculum loads [15]. Similar mortality levels have been observed in field seed bed microcosm experiments using cores from sites with moderate to high disease levels based on densities of killed seeds in the seed bank (Meyer, unpublished data). Trial experiments determined that the talc had no effect on either seeds or pathogen. For each replicate, seeds and inoculum were placed in a small vial and vortexed to coat the seeds. Dry weight of the conidial-talc inoculum was scaled to the size of the seed but was sufficient to saturate seed surfaces with inoculum. Inoculated seeds were then placed in Petri dishes on moist germination blotters and incubated at 20°C under a 12-h light/dark cycle (cool white fluorescent light). Dishes were watered as needed. Seeds were monitored for mortality (presence of stromata >1mm on ungerminated seeds), and germination (presence of >1mm radicle) on days 2, 4, 7, 11, 14, 21, and 28. After day 28, all remaining ungerminated seeds were evaluated for viability using a cut test, i.e., an evaluation of longitudinally bisected seeds [30]. Seeds with an intact embryo were scored as viable. Proportion of seeds killed, seeds germinated, and seeds ungerminated but viable were calculated as the fraction of total initially viable seeds in each replicate. No mortality due to other seed pathogens was observed.

Arcsine square root transformed data for each reciprocal inoculation experiment were first analyzed using mixed model analysis of variance (SAS 9.4 Proc Mixed) for a nested design, with host and pathogen population as fixed main effects and pathogen strain nested within pathogen population as the random effect. We then carried out variance components analysis by host species for each experiment (SAS 9.4 Proc Nested) to evaluate the contribution of the random variable strain nested within pathogen population to the experimental outcome on each host. Finally, we used correlation analysis to test whether pathogenicity at the strain level was positively or negatively correlated between hosts in each reciprocal inoculation experiment, that is, whether mortality in response to inoculation by the range of strains from each pair of host species was correlated across host species.

### Host resistance vs. host tolerance experiments

Studies of host resistance and tolerance as a function of dormancy status and inoculum load were confined to the four species for which data were not already available (*B*. *diandrus*, *B*. *rubens*, *B*. *arvensis*, *T*. *caput-medusae;* for *B*. *tectorum* see [6, 15]). The experimental design for each of the four host species included two dormancy treatments (dormant and nondormant seeds), three inoculum densities (control, low, and high), two strains, and eight replicates of 25 seeds per treatment combination, for a total of 384 experimental units and 9,600 seeds. The inoculum loads were chosen based on mortality levels on dormant seeds observed at these loads in previous work with *B*. *tectorum* [15].

Seeds from -4°C and 35°C storage were used in the dormant and nondormant seed treatments, respectively. Two strains were selected at random from the set of strains available for each pathogen population and used to inoculate seeds of the host of origin. The three inoculum densities achieved with sterile talc as described earlier were high (1:200), low (1:3200), and control (100% sterile talc). Seeds and inoculum were placed in 4-ml glass vials and shaken vigorously.

For each treatment combination, eight replicates of 25 inoculated seeds were placed in Petri dishes (100 x 15 mm) on moist germination blotters (Anchor Paper, St. Paul, MN, USA) and subjected to the same experimental regime and data collection schedule as in the experiment described earlier, with one exception. Germinated seeds were left in the Petri dishes with clipped coleoptiles and monitored for the development of stromata, making it possible to quantify infection of germinated seeds [15]. Seeds exhibiting stromatal development were immediately removed from the Petri dishes in order to prevent secondary infection. Proportion of seeds killed, germinated but infected (exhibiting stromatal development), germinated and uninfected, and ungerminated but viable were calculated as fraction of initially viable seeds in each replicate. No mortality due to other seed pathogens was observed.

The data set for each host species was analyzed separately. Proportional data were arcsine square root transformed to improve homogeneity of variance prior to analysis. Mixed model analysis of variance was carried out using SAS Proc Mixed (SAS 9.4) with dormancy status and inoculum level as fixed main effects and strain as the random effect. The control inoculum treatment in all cases resulted in essentially no disease expression and was not included in the formal analysis.

## Results

### Pathogen population genetic structure

Population genetic analyses using SSR markers and ITS sequence divergence markers for the five pathogen populations used as inoculum sources in the cross-inoculation experiments yielded very similar results. Analysis of Molecular Variance (AMOVA) of each data set without additional structure showed similar levels of among- versus within-population variance, with most of the variance distributed within populations (Table 2). Among-population variance also contributed significantly for both marker systems (17.5% for SSR’s and 21.3% for ITS). The population structure that showed the highest among-group variance was one that included pathogen populations from the three host species belonging to *Bromus* Section Genea (*B*. *tectorum*, *B*. *rubens*, and *B*. *diandrus*) in one group and the remaining pathogen populations (from *B*. *arvensis* and *T*. *caput-medusae*) in a second group. This among-group differentiation accounted for 20.2% of total variance in the SSR AMOVA and 15.7% in the ITS AMOVA, while among-population variance within groups was reduced to 3.8% and 10.5%, respectively.

**Table 2:** Analysis of Molecular Variance (AMOVA) for populations of the seed pathogen *P*. *semeniperda* from five weedy annual grass hosts based on SSR’s (7 loci) and ITS.

Genetic Structure		Microsatellite (SSR) Analysis		ITS Analysis	
	d.f.	Variance Component	% of Variance	Variance Component	% of Variance
***Study Populations***					
**No Structure**					
Among-populations	4	0.3378	17.51	0.0897	21.32
Within-populations	187	1.5909	82.49	0.3311	78.68
Host Section Genea vs. Other*					
Among-groups	1	0.4241	20.24	0.0707	15.74
Among pops wi. groups	3	0.0804	3.84	0.0473	10.53
Within-populations	187	1.5909	75.92	0.3311	73.73
Study Populations Plus Comparison Populations**					
**Grouped Geographically**					
Among-regions	3	--------	--------	0.0124	3.16
Among-pops wi. regions	4	--------	--------	0.0618	15.31
Within-populations	240	--------	--------	0.3205	81.53
**Grouped by Host Species**					
Among-species	4	--------	--------	0.0643	15.88
Among-pops wi. species	3	--------	--------	0.0199	4.92
Within-populations	240	--------	--------	0.3206	79.20

*Section Genea includes *B*. *tectorum*, *B*. *rubens*, and *B*. *diandrus*. Other includes *B*. *arvensis* and *T*. *caput-medusae*.

** Comparison populations are pathogen populations from *B*. *tectorum* collected in the same three geographic regions as pathogen populations from alternate annual grass hosts (Interior Pacific Northwest—Northern, Snake River Plains—Central, and Southern Utah—Southern). The fourth region, Northern Utah, is the source of the study population from *B*. *tectorum*. No SSR data were available for comparison populations.

**Fig 2:**
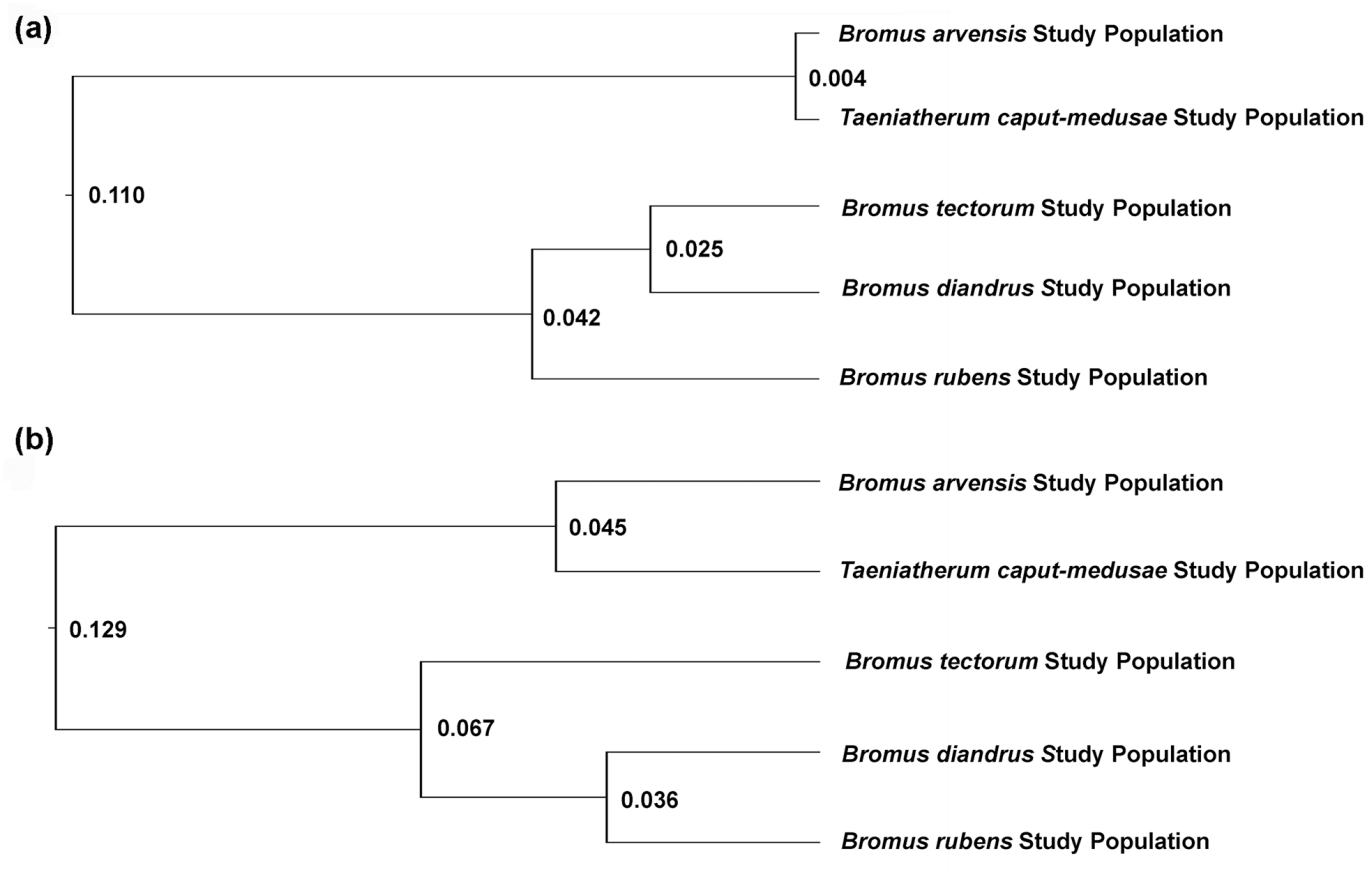
Cluster dendrograms generated by UPGMA in Neigbor (PHYLIP) using pairwise F_ST_ distance matrices from the Arlequin analysis for pathogen study populations from five weedy annual grass species based on (a) microsatellite (SSR) marker data and (b) ITS marker data. Node labels indicate genetic distance.

Cluster analysis based on pairwise F_ST_ supported the genetic division of the pathogen populations into two groups (Fig 2). Based on SSR’s, the pathogen populations from *B*. *arvensis* and *T*. *caput-medusae* were remarkably similar. They were well-differentiated from the three pathogen populations from *Bromus* Section Genea, which formed a looser cluster (Fig 2a). The ITS cluster dendrogram was very similar, with the populations from the three closely related host species well-differentiated from the other two populations (Fig 2b). In this case the relationship between populations from *B*. *arvensis* and *T*. *caput-medusae* did not appear so close. In the pathogen group on Section Genea hosts, the order of the three pathogen populations was switched, with the *B*. *tectorum* population as the outlier instead of *B*. *rubens*. These differences within the Section Genea pathogen population group were small, and even the division between the two major groups of pathogen populations occurred at a relatively short genetic distance (0.110 for the SSR analysis and 0.129 for the ITS analysis). This reflects of the fact that most genetic variation was contained within populations for both marker systems.

**Fig 3:**
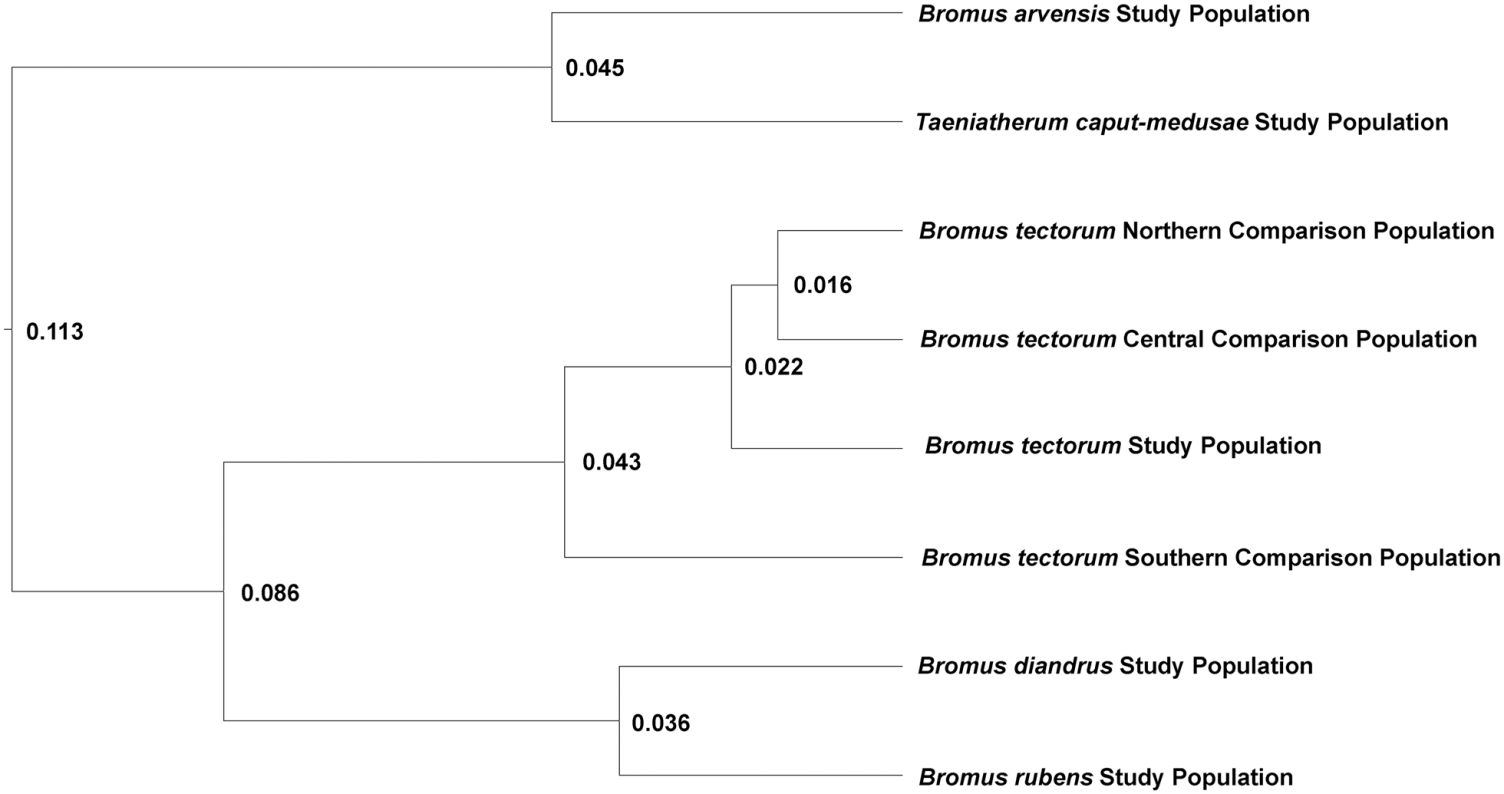
Cluster dendrogram generated by UPGMA in Neighbor (PHYLIP) using a pairwise F_ST_ distance matrix from the Arlequin analysis based on ITS sequence divergence for pathogen study populations from five weedy annual grass species along with comparison pathogen populations from *B*. *tectorum* for each region where pathogen study collections were obtained for the other four weedy annual grass species (*Bromus arvensis* pathogen population– Northern Region; *Taeniatherum caput-medusae* pathogen population Central Region; *Bromus diandrus* and *Bromus rubens* pathogen populations –Southern Region). Node labels represent genetic distance.

AMOVA was also used to test the alternative hypotheses of geographic versus among-host genetic differentiation. If geographic differentiation were more important than differentiation on different host species, then AMOVA with region as the group in the genetic structure would result in much of the among-population variation explained as among-region variation. If host differentiation were more important, then host species as the group in the genetic structure of the AMOVA would account for much of the among-population variation. It was clear from the results that the differentiation we observed in the study populations did not have a geographic basis (Table 2). Variation among regions accounted for only 3.2% of the variance, while 15.3% was still explained by the among-population within-region variance component. In the alternate analysis, the among-host-species variance component accounted for 15.8% of the variation, while the among-population within-host-species component (essentially the differences among the four populations from *B*. *tectorum* from different regions) accounted for only 4.9%. Cluster analysis based on the F_ST_ matrix that included the study populations and the comparison populations on *B*. *tectorum* from different regions also indicated that the observed differentiation was among host species rather than among regions (Fig 3).

These results support the conclusion that pathogen populations from geographically separated populations of a single host species (*B*. *tectorum*) are more similar to each other than pathogen populations from different host species collected in closer geographic proximity. This makes it likely that the differences among pathogen populations in this study could be attributed to genetic differentiation on different hosts.

We also examined gene diversity measured as expected heterozygosity across loci for each of the five pathogen study populations (Table 3). As would be expected based on the large within-population component of variance in the AMOVAs, gene diversity was generally quite high. For the SSR marker system, differences among the seven loci in the degree of polymorphism observed resulted in high standard deviations for expected heterozygosity, but mean values did not vary greatly among pathogen populations (range 0.436 to 0.529). Gene diversity was higher and among-population variation was greater for the ITS marker, with study populations on *B*. *tectorum* and *B*. *diandrus* showing the lowest values and those on *B*. *arvensis* and *T*. *caput-medusae* showing the highest (range 0.583 to 0.745).

**Table 3:** Gene diversity (mean expected heterozygosity) for five *P*. *semeniperda* populations from different weedy annual grass hosts based on two molecular marker systems (SSR’s and ITS sequence divergences).

Host Species	Sample Size*	Microsatellite (SSR) Gene Diversity	ITS Gene Diversity**
*Bromus tectorum*	28	0.529±0.123	0.583
*Bromus rubens*	46	0.436±0.240	0.652
*Bromus diandrus*	40	0.476±0.187	0.604
*Bromus arvensis*	36	0.529±0.191	0.722
*Taeniatherum caput-medusae*	42	0.501±0.223	0.745

* Heterokaryotic strains with two alleles at a locus were treated as two strains with identical ITS and SSR haplotypes except at polymorphic SSR loci, resulting in an increase in effective sample size for each pathogen population. ITS loci were never polymorphic even within heterokaryotic strains.

**Standard deviations for SSR’s based on n = 7 loci. No standard deviation is included for ITS because n = 1.

### Cross-inoculation experiments

**Fig 4:**
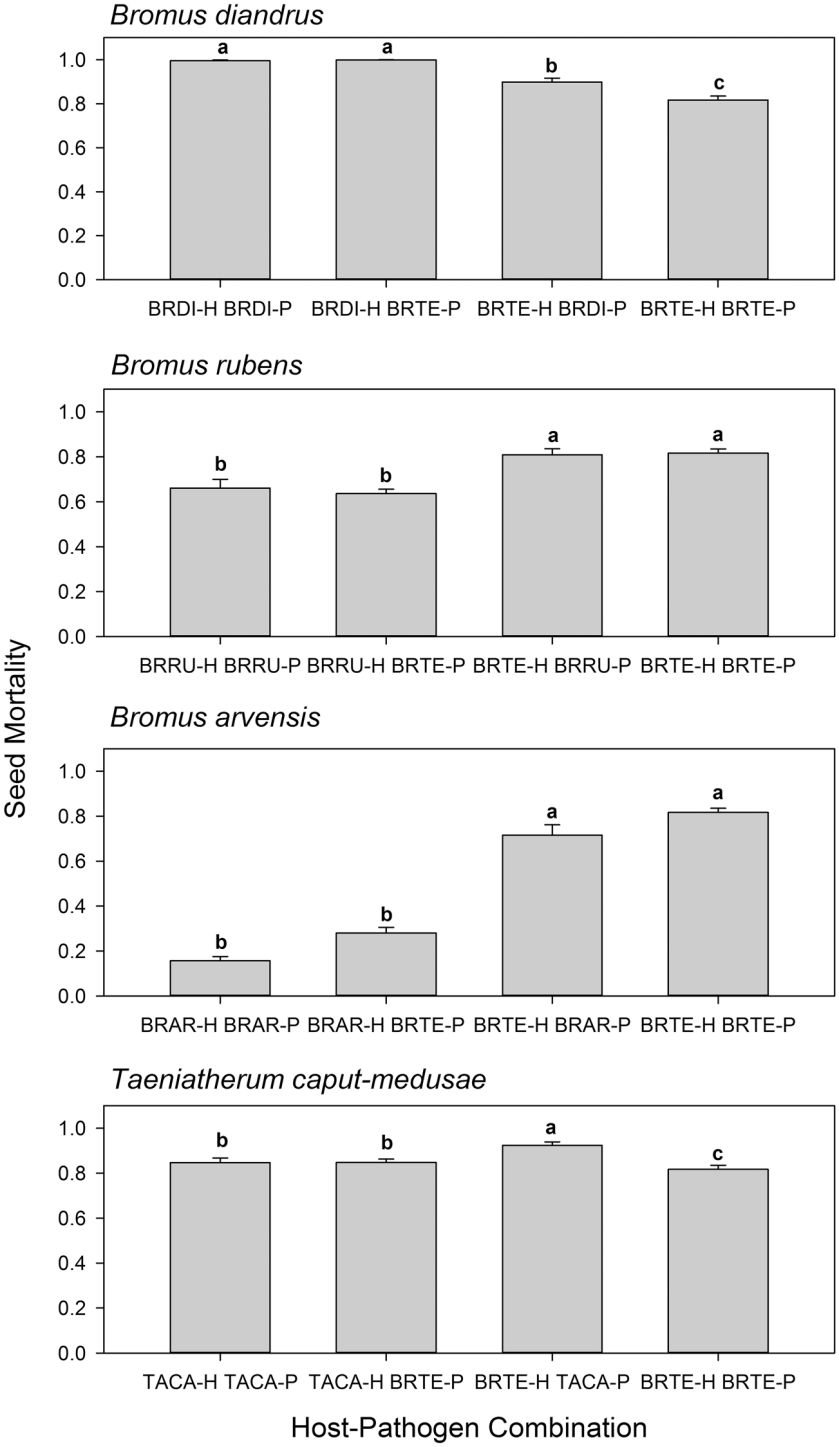
Mean host seed mortality in four cross-inoculation trials in which pathogen strains from *B*. *tectorum* and an alternate host were inoculated reciprocally onto both hosts. Order of means presentation in each panel: alternate host inoculated with alternate pathogen population, alternate host inoculated with *B*. *tectorum* pathogen population, *B*. *tectorum* host inoculated with alternate pathogen population, *B*. *tectorum* host inoculated with *B*. *tectorum* pathogen population. For each cross-inoculation trial (alternate host identified in panel title), means headed by different letters are significantly different at P<0.05 based on LSmeans separations from analysis of variance. Error bars represent standard error of the mean. See Table 4 for statistical analysis.

Cross-inoculation experiments with pathogen populations from *B*. *tectorum* and other annual grass hosts showed contrasting patterns (Table 4; Fig 4). In *B*. *diandrus* x *B*. *tectorum* cross-inoculations, *B*. *diandrus* was highly susceptible, suffering >99% mortality regardless of pathogen population, whereas *B*. *tectorum* was slightly less susceptible overall and showed significantly lower mortality when inoculated with its own pathogen strains (Fig 4a). These results provide weak evidence of local maladaptation for the pathogen population from *B*. *tectorum* in that it caused higher mortality on *B*. *diandrus* than on its own host and also caused lower mortality than the *B*. *diandrus* pathogen population on *B*. *tectorum*, its host of origin. These differences, though significant, were small.

**Table 4:** Analysis of variance (SAS Proc Mixed) for reciprocal inoculation trials using pathogen populations from four annual grass weeds. Each pathogen population was inoculated onto its own host seeds and onto *Bromus tectorum* seeds. A fifth pathogen population from *Bromus tectorum* was inoculated onto its own host seeds and onto seeds of each of the four alternate hosts. Each pathogen population was represented by six strains (four strains for *B*. *arvensis*). The data were analyzed as four separate experiments with host species and pathogen population as the fixed main effects and strain nested with pathogen population as the random effect.

Alternate Host	Effect	d. f.	F	P
*Bromus diandrus*	Host Species	1,178	297.35	<0.0001
	Pathogen Population	1,10	1.96	0.1918
	Host x Pathogen Population	1,178	22.03	<0.0001
	Strain(Pathogen Population)		1.97*	0.0244
*Bromus rubens*	Host Species	1,178	56.30	<0.0001
	PathogenPopulation	1,10	0.13	0.7241
	Host x Pathogen Population	1,178	1.50	0.2226
	Strain(Pathogen Population)		2.09*	0.0183
*Bromus arvensis*	Host Species	1,148	43.17	<0.0001
	Pathogen Population	1,8	2.09	0.1862
	Host x Pathogen Population	1,148	1.11	0.2934
	Strain(Pathogen Population)		1.76*	0.0396
*Taeniatherum caput-medusae*	Host Species	1,178	6.49	0.0117
	Pathogen Population	1,10	2.28	0.1618
	Host x Pathogen Population	1,178	17.36	<0.0001
	Strain(Pathogen Population)		2.06*	0.0198

*Test statistic is Wald Z test of the significance of the variance contribution of the covariance parameter strain (pathogen origin) from mixed model analysis of variance.

In *B*. *rubens* and *B*. *tectorum* cross-inoculations, the observed pattern clearly supported the hypothesis of differential host resistance, with *B*. *rubens* more resistant than *B*. *tectorum* to pathogen populations from both *B*. *tectorum* and *B*. *rubens* (Table 4, Fig 4b). There was no evidence of a difference between pathogen populations overall and no significant interaction between pathogen population and host species. There was thus no evidence for either host specialization or local maladaptation.

In *B*. *arvensis* x *B*. *tectorum* cross-inoculations, seed mortality differed significantly between the two hosts, with *B*. *arvensis* showing substantially lower seed mortality than *B*. *tectorum* (Table 4, Fig 4c). Apparently higher mortality on both hosts when inoculated with the *B*. *tectorum* pathogen population suggested a possible difference in pathogenicity, but this proved not to be statistically significant, possibly because of reduced statistical power due to the smaller number of strains from *B*. *arvensis*. This interaction therefore reduced to a simple case of difference in host resistance similar to *B*. *rubens*.

In cross-inoculations with pathogen populations from *T*. *caput-medusae* and *B*. *tectorum*, there was a significant host species-pathogen population interaction (Table 4, Fig 4d). The two pathogen populations performed essentially identically on the *T*. *caput-medusae* host, whereas on the *B*. *tectorum* host, the *T*. *caput-medusae* pathogen population caused significantly higher mortality than the *B*. *tectorum* pathogen population. Strains from both *B*. *tectorum* and *T*. *caput-medusae* exhibited slight local maladaptation in that they caused significantly higher mortality on the non-local host than on the host of origin. Although this result represents the pattern for local maladaptation, the differences were again very small.

The universal lack of a significant pathogen population main effect in the analysis of variance (Table 4) is likely due to the large error term for this effect, namely the term attributable to strain nested within pathogen population. Variance component analysis (SAS Proc Nested) was performed for each host in each cross-inoculation experiment, so that the effects of among-strain variation could be evaluated for each case (Table 5).

**Table 5:** Variance component analysis (SAS 9.4 Proc Nested) for pathogen populations and strains on each host in inoculations with pathogen populations from four annual grass weeds reciprocally inoculated onto their own hosts and onto *Bromus tectorum*.

Reciprocal Inoculation	d.f.	F	P	Variance %
*Bromus diandrus* Reciprocal Inoculation				
*Bromus diandrus* host				
Pathogen Population	1	0.90	0.3655	0
Strain(Pathogen Population)	10	1.52	0.1456	6.1
Error	84			93.9
*Bromus tectorum* host				
Pathogen Population	1	2.57	0.1401	14.4
Strain(Pathogen Population)	10	12.73	<0.0001	50.9
Error	84			34.7
*Bromus rubens* Reciprocal Inoculation				
*Bromus rubens* host				
Pathogen Population	1	0.29	0.6012	0
Strain(Pathogen Population)	10	13.85	<0.0001	61.6
Error	84			38.4
*Bromus tectorum* host				
Pathogen Population	1	0	0.9568	0
Strain(Pathogen Population)	10	6.93	<0.0001	42.6
Error	84			57.4
*Bromus arvensis* Reciprocal Inoculation				
*Bromus arvensis* host				
Pathogen Population	1	3.95	0.0821	18.5
Strain(Pathogen Population)	8	4.12	0.0005	22.9
Error	70			58.6
*Bromus tectorum* host				
Pathogen Population	1	0.62	0.4554	0
Strain(Pathogen Population)	8	8.18	<0.0001	47.3
Error	70			52.7
*Taeniatherum caput-medusae* Reciprocal Inoculation				
*Taeniatherum caput-medusae* host				
Pathogen Population	1	0.11	0.7416	0
Strain(Pathogen Population)	10	5.79	<0.0001	37.5
Error	84			62.5
*Bromus tectorum* host				
Pathogen Population	1	6.10	0.0331	30.8
Strain(Pathogen Population)	10	7.18	<0.0001	31.5
Error	84			37.7

Differences among strains within pathogen populations in the case by case analysis were highly significant in all but the case of *B*. *diandrus*, in which all strains caused uniformly high mortality. Differences between pathogen populations were generally not significant (Table 5), a result similar to that obtained from the overall analysis for each cross-inoculation (Table 4). Among-strain variation within populations accounted for 23–62% of the total variance and 51–100% of model variance in the seven of eight cases in which among-strain differences were significant (Table 5).

**Fig 5:**
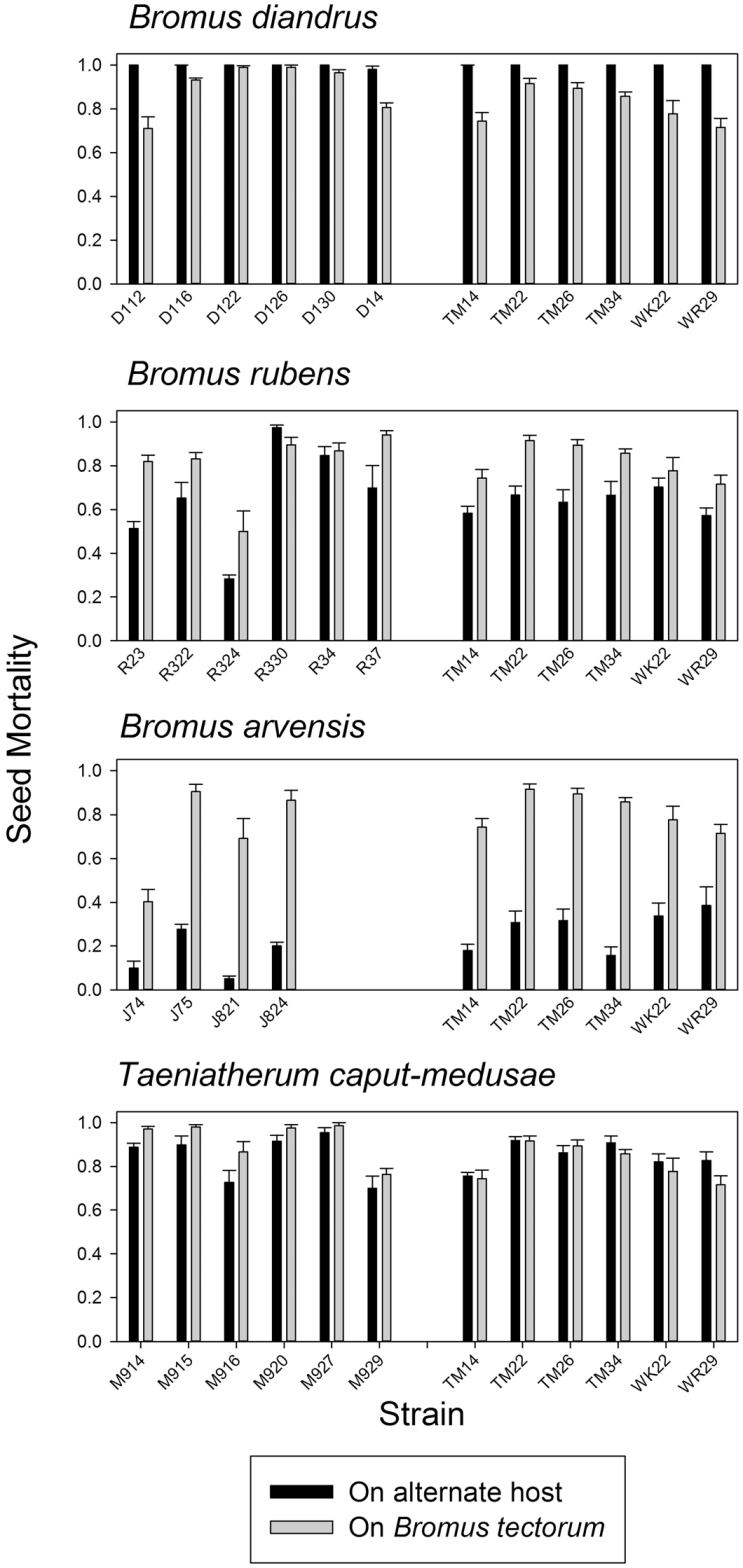
Mean host seed mortality following inoculation with each strain in four cross-inoculation trials in which six pathogen strains from *B*. *tectorum* (right hand side) and six strains from an alternate host (four strains from *B*. *arvensis*; left hand side) were inoculated reciprocally onto both hosts. Alternate hosts are identified in panel title. Error bars represent standard error of the mean. See Table 5 for variance component analysis by host for each cross-inoculation.

Major among-strain variation in pathogenicity was readily observable in plots of seed mortality by strain for each host in cross-inoculation trials (Fig 5). A trend evident in the plots of seed mortality by strain on each pair of hosts was that strains that caused higher mortality on one host also seemed to cause higher mortality on the other host. This trend would argue against either host specialization or local maladaptation, but instead would suggest that strains within populations vary in aggressiveness, and that this variation is expressed similarly on both hosts. Correlation analysis generally supported this conclusion or at least provided no evidence to the contrary. Strain-specific mortality percentages were significantly positively correlated between hosts for *B*. *rubens* and *B*. *tectorum* cross inoculations (r = +0.756, n = 12, p = 0.0044) and for *T*. *caput-medusae* and *B*. *tectorum* cross inoculations (r = +0.725, n = 12, p = 0.0076). A similar but non-significant positive correlation was observed for *B*. *arvensis* and *B*. *tectorum* cross inoculations (r = +0.486, n = 10, p = 0.154). No correlation was observed in the *B*. *diandrus* and *B*. *tectorum* cross inoculations because of lack of variability in seed mortality on *B*. *diandrus* (r = +0.187, n = 12, p = 0.561). If either host specialization or local maladaptation were the ascendant process at the strain level, negative correlations in mortality percentages on the two hosts would be the predicted outcome, but this was not the case. This leads to the conclusion that *P*. *semeniperda* is a true generalist pathogen that lacks significant host specialization on any of the weedy annual grasses included in this study.

### Host resistance versus host tolerance experiments

**Fig 6:**
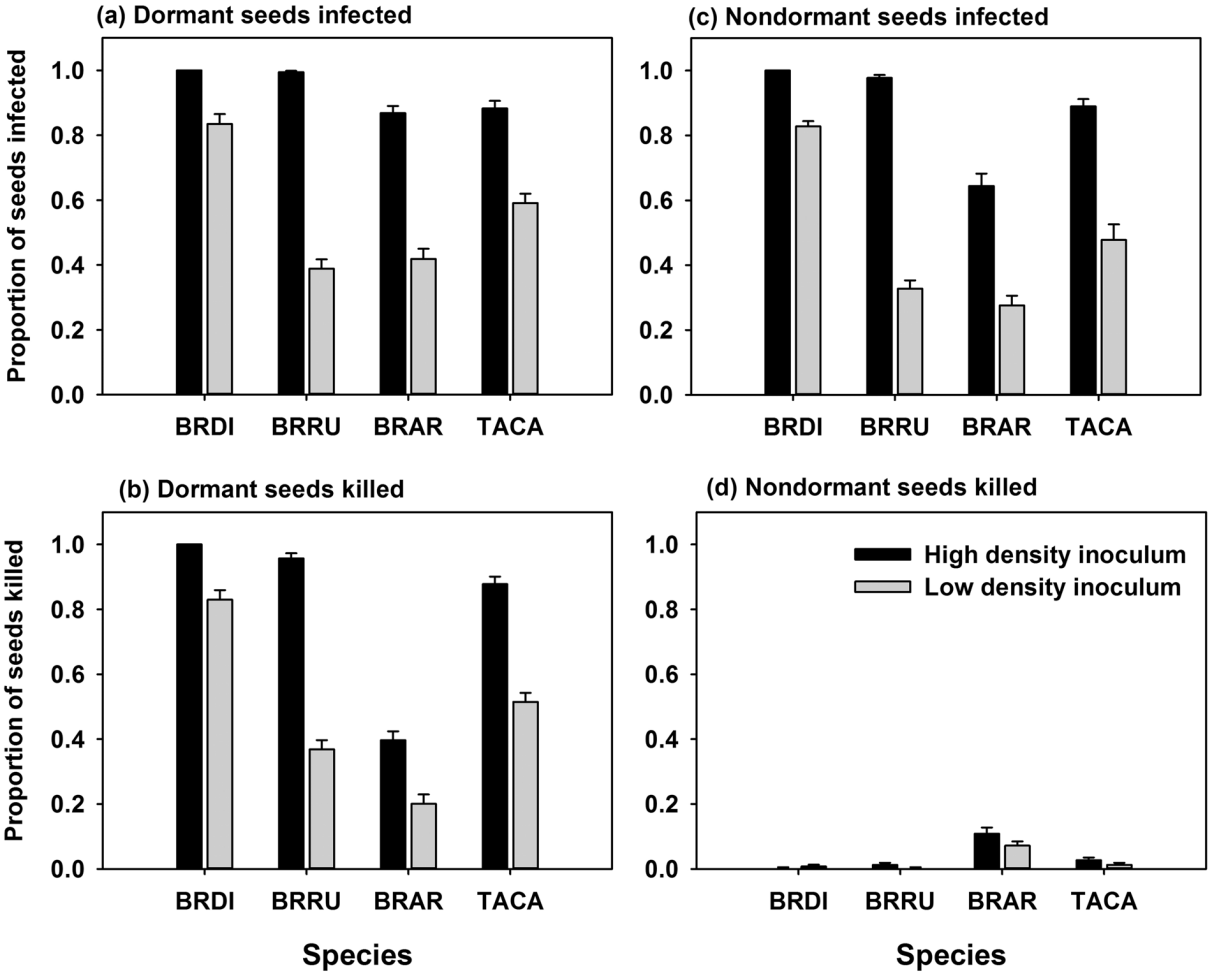
Proportion of: (a) dormant seeds infected, (b) dormant seeds killed, (c) nondormant seeds infected, and (d) nondormant seeds killed by *Pyrenophora semeniperda* for seeds of four host species at two inoculum levels. (Host species included: *Bromus diandrus* (BRDI), *Bromus rubens* (BRRU), *Bromus arvensis* (BRAR), and *Taeniatherum caput-medusae* (TACA). Error bars represent standard error of the mean. See Table 6 for statistical analysis.

Results of inoculation experiments with dormant and nondormant seeds supported the hypothesis that seeds of weedy annual grass hosts would exhibit high susceptibility to infection by *P*. *semeniperda* whether dormant or nondormant, but would be intolerant of infection (i.e., killed) only when dormant (Table 6, Fig 6). For all four species, infection levels as evidenced by sporulation on seeds were high, especially at the high inoculum level, whether seeds were dormant (Fig 6a) or nondormant (Fig 6c), whereas high seed mortality was the outcome only on dormant seeds (Fig 6b). Very few nondormant seeds were killed prior to germination (Fig 6d). This is very similar to the pattern observed earlier for dormant versus nondormant seeds of *B*. *tectorum* [6, 15]. These results support the hypothesis that infection levels and mortality levels on dormant seeds are generally similar at a given inoculum load because dormant seeds are intolerant to infection, whereas infection levels on nondormant seeds are much higher than mortality levels because nondormant seeds are tolerant to infection. Seeds of all four species exhibited at least some degree of primary dormancy when recently harvested and lost dormancy through dry after-ripening at summer temperatures, and patterns of mortality were directly related to dormancy status.

**Table 6:** Mixed model analysis of variance for experiments in which dormant and nondormant host seeds of four weedy grasses were inoculated with two strains of their own pathogen populations at two inoculum levels. Dormancy status and inoculum level were the fixed main effects while strain was the random effect in each analysis. The dependent variables were proportion of seeds infected and proportion of seeds killed.

Species and Effects		Seed Infection		Seed Mortality	
	d.f.	F	P	F	P
*Bromus diandrus*					
Dormancy status	1,59	0.50	0.4802	3237.12	<0.0001
Inoculum level	1,59	243.55	<0.0001	65.83	<0.0001
Dormancy status x inoculum level	1,59	0.50	0.4802	78.68	<0.0001
*Bromus rubens*					
Dormancy status	1,59	6.15	0.0160	1171.40	<0.0001
Inoculum level	1,59	1111.60	<0.0001	198.50	<0.0001
Dormancy status x inoculum level	1,59	0	0.9888	159.45	<0.0001
*Bromus arvensis*					
Dormancy status	1,59	50.67	<0.0001	74.13	<0.0001
Inoculum level	1,59	208.80	<0.0001	17.57	<0.0001
Dormancy status x inoculum level	1,59	5.37	0.0241	6.83	0.0114
*Taeniatherum caput-medusae*					
Dormancy status	1,59	2.57	0.1143	887.71	<0.0001
Inoculum level	1,59	125.81	<0.0001	64.99	<0.0001
Dormancy status x inoculum level	1,59	1.89	0.1742	37.91	<0.0001

Inoculum level had a significant effect on both seed infection and mortality for all four species (Table 6, Fig 6). The effect was least pronounced for *B*. *diandrus*, which had high infection and dormant seed mortality even at the low inoculum level. It was most pronounced for *B*. *rubens*, which showed a major drop in infection and dormant seed mortality at the lower level.

*Bromus arvensis* showed an anomalous pattern relative to the other three species, with relatively low mortality even when dormant. This was likely because this collection of *B*. *arvensis* had relatively low dormancy even prior to after-ripening. Its response resembled the response of partially after-ripened seeds rather than truly dormant seeds, with many susceptible seeds escaping through germination. This anomalous response was also observed in the cross-inoculation study, in which mortality on *B*. *arvensis* was low regardless of pathogen origin.

## Discussion

We found no evidence of host specialization for *P*. *semeniperda* on the seeds of five winter annual grass species included in this study. These species share many life history features that make them ideal hosts for this pathogen. Seeds are produced in large quantities, with many thousands per square meter in the autumn seed bank even after post-dispersal seed predation. Most seeds escape through rapid germination in the fall, ensuring that the pathogen will have seeds to infect in future years. But some sporulation on germinated seeds enables pathogen persistence even under conditions when most or all seeds germinate in the first germination-triggering rainfall, even though pathogen fitness is likely reduced on nondormant seeds because seedlings usurp resources that would be available to the pathogen on dormant seeds. Seeds may be killed if rains occur in summer prior to complete dormancy loss [31], or if inadequate autumn precipitation creates water stress conditions that make the seeds more vulnerable to attack [32]. Inadequate fall rains may also leave a large fraction of viable seeds to overwinter. *Bromus tectorum* seeds can be rendered secondarily dormant under winter conditions [33], providing the pathogen with vulnerable prey in spring, and reports indicate that the other winter annuals in this study exhibit similar secondary dormancy [34–36].

The host species in this study included only those with a strong presence of *P*. *semeniperda* in field seed banks, as field-killed seeds were the source of the strains from each host. Many species that show high susceptibility to this pathogen in laboratory inoculation trials do not have high densities of killed seeds in field seed banks [15]. For example, it was not possible to include native cool-season grasses in cross-inoculation trials because very few or no *P*. *semeniperda*-killed seeds could be found in their seed banks. Similarly, the exotic winter annual grass *Ventenata dubia* was found to be susceptible to the pathogen in laboratory inoculations [15], but no pathogen-killed seeds were found in field seed banks of this species.

While this pathogen can attack a wide range of grass hosts when applied at very high inoculum loads in laboratory pathogenicity trials, it is much less successful on native grass species at inoculum loads more characteristic of field conditions [15]. We have also shown in field experiments that native grass seeds killed by this pathogen tend to be the slowest-germinating seeds in a population, and that these seeds generally fail to emerge due to other causes in the absence of artificially increased pathogen inoculum loads [7].

It appears that *P*. *semeniperda* is well-adapted to attack a suite of grass species with closely similar seed biology, and that host specialization is not a necessary component of high fitness across this suite of species. It is possible that strains originating from hosts with very different seed biology, e.g., the native perennial grass *Achnatherum hymenoides*, would exhibit more host specialization in cross-inoculation trials. However we have not demonstrated unequivocally that this pathogen species includes strains that originated on any North American native grass hosts, in spite of our considerable effort to examine this question.

Contrary to earlier reports [37], this pathogen is common in the seed banks of weedy annual grasses, including *B*. *tectorum* and *T*. *caput-medusae*, in the native Eurasian range [38]. Population genetic structure of strains on B. *tectorum* across the invaded North American range suggests that they were introduced along with host seeds from Eurasia [22]. The pathogen is known to disperse its conidia onto the undispersed seeds of host plants [39], making it likely that specific strains will travel together with host seeds during both short and long-distance dispersal. This founder effect could explain why pathogen populations were somewhat genetically differentiated on different hosts. The differentiation was weak because there is no host specialization, so that conidia can succeed in infecting whatever annual grass host seeds they encounter. This process of blurring of the genetic differences due to founder effects could be quite slow, however, as the pathogen is soilborne and has no special adaptations for dispersal independent of host seeds.

Among-strain variation in aggressiveness on dormant seeds was observed in this study on all but the most highly susceptible host, *B*. *diandrus*. This among-strain variation in the ability to cause dormant seed mortality at relatively low inoculum loads is part of a larger suite of variable adaptive traits exhibited by this pathogen on *B*. *tectorum* and is specifically associated with variation in mycelial growth rate [24, 26]. Fast-growing strains are more likely to kill dormant seeds at low inoculum loads, whereas slow-growing strains are more likely to cause nondormant seed mortality at high inoculum loads. This effect on nondormant seeds occurs because slow growth is associated with the high toxin production necessary to disable and kill germinating seeds [26]. At high inoculum loads, high mortality on dormant seeds is the outcome regardless of strain variation. In experiments with dormant vs. nondormant seeds in this study, among-species differences in dormant seed resistance were much more evident at the lower inoculum level. As expected, the high inoculum level caused near-complete mortality on dormant seeds (except for *B*. *arvensis*, discussed above). The intermediate inoculum level used in the cross-inoculation experiment was thus possibly too high to elicit major differences among strains in dormant seed mortality. In spite of this, however, there was generally a positive correlation between seed mortality on *B*. *tectorum* and on the alternate host, indicating that the effect of inoculum load did not mask among-strain differences in aggressiveness on dormant seeds.

## Supporting Information

S1 Supporting Information Data sets for each analysis included in the results. (XLSX)
